# Preoperative Advanced Lung Cancer Inflammation Index and Acute Kidney Injury After Adult Cardiac Surgery: A Retrospective Cohort Study

**DOI:** 10.3390/jcdd13100490

**Published:** 2026-09-30

**Authors:** Haoran Gan, Ruoyu Zhang, Rui Fan, Li Yin, Shengjie Ning, Junzhe Wang, Dongming Liu, Wen Chen, Xin Chen, Zhibing Qiu

**Affiliations:** 1 Department of Thoracic and Cardiovascular Surgery, Nanjing First Hospital, Nanjing University of Chinese Medicine, Nanjing 210006, China; 2 Department of Thoracic and Cardiovascular Surgery, Nanjing First Hospital, Nanjing Medical University, Nanjing 210006, China

**Keywords:** acute kidney injury, adult cardiac surgery, advanced lung cancer inflammation index, albumin, inflammation, nutrition

## Abstract

Acute kidney injury (AKI) is common after cardiac surgery. We assessed the association between the preoperative advanced lung cancer inflammation index (ALI) and postoperative AKI. This retrospective cohort included adults undergoing cardiac surgery from 2 January 2019 to 16 March 2026. The primary outcome was the first in-hospital AKI recorded within 168 h using stored Kidney Disease: Improving Global Outcomes creatinine and kidney-replacement-therapy classifications. Among 11,890 adults, AKI was recorded in 3380 (28.4%). Each ALI doubling was associated with a lower adjusted AKI rate (hazard ratio, 0.83; 95% confidence interval, 0.80–0.86; *p* < 0.001); the fixed-horizon risk ratio was 0.86 (95% confidence interval, 0.83–0.88; *p* < 0.001). Adjustment for available preoperative shock, resuscitation, intubation, infective endocarditis, acute aortic disease, and catecholamine indicators attenuated the hazard ratio to 0.90 (95% confidence interval, 0.87–0.94). Association magnitude differed across mutually exclusive procedure categories (interaction *p* < 0.001). The coefficient constraints imposed by ALI were rejected, and the positive BMI association persisted with flexible modeling. Lower ALI was associated with more recorded AKI, but differential monitoring, residual confounding, and unsupported component constraints limit interpretation of the composite.

## 1. Introduction

Acute kidney injury (AKI) is a frequent complication after cardiac surgery and is associated with prolonged intensive care, kidney replacement therapy, early death, and subsequent chronic kidney disease [1,2]. Prevention guidance therefore emphasizes preoperative recognition of susceptibility, avoidance of nephrotoxins, and structured perioperative kidney-protection strategies [3,4]. The risk of cardiac surgery-associated AKI reflects baseline kidney reserve, procedural complexity, hemodynamic disturbance, ischemia–reperfusion, inflammation, cardiopulmonary bypass, and postoperative complications [1,4].

The advanced lung cancer inflammation index (ALI) is calculated as body mass index multiplied by serum albumin and divided by the neutrophil-to-lymphocyte ratio [5]. Although first developed in metastatic lung cancer, ALI has subsequently been studied as an inflammation-nutrition marker in nonmalignant and cardiovascular settings [6,7]. Its possible relevance to cardiac surgery is not cancer-specific: the index combines systemic inflammatory burden with nutritional and physiologic reserve, both of which may influence susceptibility to cardiopulmonary bypass inflammation, ischemia–reperfusion, and perioperative hemodynamic stress [1,4]. The composite nevertheless imposes a fixed direction on each component: higher body mass index and albumin increase ALI, whereas higher NLR decreases it. To our knowledge, no previous study has specifically evaluated preoperative ALI in a broad adult cardiac-surgery cohort with postoperative AKI as the primary outcome.

Preoperative risk assessment commonly considers demographic characteristics, kidney function, ventricular function, comorbidity, and procedure type. Routine blood measurements may also capture inflammatory and nutritional vulnerability. Blood-cell-derived indices, including the neutrophil-to-lymphocyte ratio, systemic immune inflammation index, systemic inflammatory response index, and aggregated index of systemic inflammation, have been examined in cardiac surgery populations [8,9,10,11,12,13]. Studies have also evaluated nutritional indices, albumin-containing ratios, fibrinogen, and iron metabolism markers in relation to postoperative AKI, although association and predictive increment are distinct questions [14,15,16,17,18].

We therefore evaluated the association between preoperative ALI and the first recorded AKI event within 168 h after adult cardiac surgery. ALI was examined continuously, by quartiles, and with flexible splines. We also assessed severe AKI, calendar-period consistency, mutually exclusive surgical strata, perioperative adjustment, selection bias, and the information carried by ALI relative to its individual components.

## 2. Materials and Methods

### 2.1. Study Design and Participants

This single-center retrospective cohort study was conducted in the Department of Thoracic and Cardiovascular Surgery, Nanjing First Hospital, Nanjing, China. Consecutive patients aged at least 18 years who underwent cardiac surgery from 2 January 2019 through 16 March 2026 were eligible. Age at surgery was taken from the corrected age field in the locked source database. To retain one index operation per patient, records were ordered by cleaned patient identifier, surgery end time, and source-row index, and the earliest operation was retained; six later operations were excluded. Combined procedures during the index operation remained eligible. We excluded 21 patients with missing age or age younger than 18 years, 155 receiving preoperative dialysis, 898 whose source outcome-observability flag was false, and 714 with a missing or nonpositive ALI component. The source flag required a valid baseline creatinine and sufficient post-6 h creatinine, kidney replacement therapy, or early endpoint documentation to classify the in-hospital 168 h outcome. Of the 898 unobservable outcomes, 814 lacked linked raw laboratory information, 73 had no postoperative creatinine or kidney replacement therapy record, and 11 lacked a valid baseline creatinine. The analytic cohort comprised 11,890 adults. All eligible records were included; no formal sample-size calculation was performed. Reporting followed the Strengthening the Reporting of Observational Studies in Epidemiology statement [19].

### 2.2. Exposure Assessment

The primary exposure was preoperative ALI, calculated as body mass index (kg/m^2^) multiplied by serum albumin (g/dL) and divided by the neutrophil-to-lymphocyte ratio [5]. Albumin was converted from g/L to g/dL. Body mass index was derived from the height and weight fields, and albumin, neutrophil count, and lymphocyte count were the values stored in the source extract’s structured preoperative fields. Their preoperative designation was inherited from the source data model; the extract did not retain a common collection timestamp or all candidate repeated tests for the four components. We could therefore verify that no postoperative field was used but could not independently reconstruct exact look-back intervals, same-day concordance, or alternative repeated-test selection rules. All components had to be present and greater than zero. ALI was log2-transformed, so the primary estimate represents the change in the recorded AKI rate for each doubling of ALI. Quartiles and natural cubic splines described the exposure distribution without an outcome-optimized cutoff.

### 2.3. Outcome Assessment

The primary outcome was the first in-hospital AKI recorded within 168 h after the documented end of surgery. The institutional source pipeline classified AKI using Kidney Disease: Improving Global Outcomes serum-creatinine and kidney-replacement-therapy criteria: an increase in creatinine of at least 26.5 μmol/L within 48 h, an increase to at least 1.5 times baseline within 7 days, or kidney replacement therapy [20]. The stored trace contained the earliest qualifying timestamp and source and the maximum stage within 168 h. Timed preoperative laboratory-information-system creatinine was selected for 11,831 patients; a validated wide-table baseline field was used for 59. The locked extract did not retain the complete creatinine-value trajectory or the upstream derivation program, so every pairwise KDIGO comparison could not be rerun independently from the distributed analysis files. Preoperative AKI was not separately phenotyped; the endpoint should therefore be interpreted as postoperative detection relative to the selected baseline, not necessarily new-onset AKI. Urine output was unavailable. Severe AKI comprised maximum stages 2 and 3. Follow-up ended at AKI, death, discharge, or 168 h, whichever occurred first. The endpoint is consequently an in-hospital recorded outcome rather than complete 168 h risk after discharge.

### 2.4. Covariates and Missing Data

The prespecified M2 variables were corrected age, sex, baseline creatinine, diabetes, hypertension, documented heart failure, and indicators for coronary artery bypass grafting, valve surgery, aortic surgery, congenital heart surgery, and heart transplantation. Heart failure was positive when either structured chronic or acute heart-failure field was recorded before surgery. The extract did not preserve whether the label came from prior history, admission assessment, or administrative coding; it was not defined by LVEF. Sensitivity analyses omitted heart failure and compared M2 with and without LVEF in the same 8995 complete cases. A post hoc preoperative clinical-state model added available indicators for cardiogenic shock, cardiopulmonary resuscitation, tracheal intubation, infective endocarditis, acute aortic dissection or intramural hematoma, and intravenous catecholamine use. Operative sensitivities included bypass and cross-clamp durations, sternotomy, repeat cross-clamping, distal venous anastomosis count, valve-position count, intra-aortic balloon pump use, and extracorporeal membrane oxygenation. Body mass index was excluded from M2 because it is an ALI component. LVEF was missing for 2895 patients; all M2 and preoperative clinical-state variables were complete.

### 2.5. Statistical Analysis

Continuous variables were summarized as mean (standard deviation) or median [interquartile range], and categorical variables as number/denominator (percentage). Baseline characteristics were presented by AKI status with standardized mean differences rather than significance tests.

Cause-specific Cox regression was used to estimate hazard ratios (HRs) and 95% confidence intervals (CIs). M0 included log2-transformed ALI; M1 additionally included age and sex; and M2 further included the remaining prespecified covariates. Death and discharge without recorded AKI were treated as censoring events for this etiologic rate estimand. A centered natural cubic spline with four degrees of freedom was fitted to log2-transformed ALI. The five resulting knots corresponded to ALI values of 0.94, 28.81, 42.30, 59.18, and 389.02, with the first and last serving as boundary knots. Likelihood-ratio tests were used to assess the overall association and nonlinearity. The proportional-hazards assumption was assessed using scaled Schoenfeld residuals for individual model terms and the full model. Because the tests for ALI and the global model indicated nonproportionality, post hoc cause-specific Cox models were fitted for 0–24, >24–72, and >72–168 h among patients who were event-free and under observation at the start of each interval.

Calendar period consistency was evaluated in an early cohort (2019–2023) and a late cohort (1 January 2024–16 March 2026), with an ALI-by-period interaction; this same-center comparison was not considered external validation. Component models added ALI, BMI, albumin, NLR, or all three components to the same clinical covariates. The constrained ALI model was compared with a free BMI–albumin–NLR model by a two-degree-of-freedom likelihood-ratio test. BMI was also modeled with a four-degree-of-freedom natural cubic spline while albumin and NLR remained in the model. The albumin–NLR analysis used three-degree-of-freedom splines and their nine-term tensor product; the interaction was compared with an additive spline model, and joint IQR contrasts were estimated with 95% CIs. These analyses described association fit rather than prediction.

Severe AKI was analyzed with multivariable binomial logistic regression using M2. The maximum 168 h KDIGO stage and total postoperative kidney replacement therapy count were also summarized. A supportive full-cohort model coded every patient without recorded stage 2 or 3 AKI as a non-event; a sensitivity model excluded death or discharge before 168 h without severe AKI.

Procedure heterogeneity was examined with eight mutually exclusive CABG, valve, and aortic combinations, stratum-specific models, an ALI-by-combined-procedure interaction, and a seven-degree-of-freedom global ALI-by-category likelihood-ratio test. The category with none of the three major-domain indicators was decomposed by congenital and transplant indicators. Operative duration and mechanical support models were treated as downstream-inclusive sensitivities because initiation times were not consistently separated. The malignancy exclusion used the structured cancer indicator; ALI-tail trimming removed values below the 0.5th and above the 99.5th percentiles; and the age sensitivity excluded two records whose corrected age differed from the earlier mapping by more than 10 years. eGFR was recalculated for every patient with the 2021 CKD-EPI creatinine equation from corrected age, sex, and baseline creatinine [21], and the eGFR ≥ 60 sensitivity was rerun.

Creatinine monitoring was summarized in nonoverlapping 0–24, >24–48, >48–72, and >72–168 h intervals. For each ALI quartile and interval, we reported the number alive, in hospital, and event-free at interval start, AKI events during the interval, measurement counts, and the proportion with at least one measurement; a second denominator retained only patients observed and event-free through interval end. Tests across the eight monitoring comparisons used the Benjamini–Hochberg procedure. Modified Poisson regression estimated the recorded 168 h AKI risk ratio with HC0 robust standard errors. The full model coded all 8510 patients without recorded AKI as non-events, including 600 whose follow-up ended before 168 h; the 41 deaths were included within those 600 because death and follow-up-end timestamps coincided. Exclusion of the 600 was treated only as a sensitivity analysis. Selection weights were estimated among 13,502 adult nondialysis index patients. Missing predictors were median-imputed with missingness indicators, predicted inclusion probabilities were bounded at 0.01–0.99, and stabilized weights were truncated at the selected cohort’s 1st and 99th percentiles. Weighted balance was assessed with standardized mean differences. All added analyses were post hoc.

During revision, the authors used ChatGPT and Codex (OpenAI OpCo, LLC, 1455 Third Street, San Francisco, CA 94158, USA; GPT-5-series models accessed through the ChatGPT web interface and Codex desktop application in September 2026) for language and structural editing, document formatting, and generation and checking of analysis code. The tools were not treated as authors and did not make final methodological or interpretive decisions. AI-assisted code was executed locally, and aggregate outputs and manuscript statements were checked against the locked data and reproducible scripts. Statistical analyses were performed using Python (versions 3.13.5 and 3.10.15) and R (version 4.5.2).

## 3. Results

### 3.1. Cohort and Baseline Characteristics

**Figure 1:**
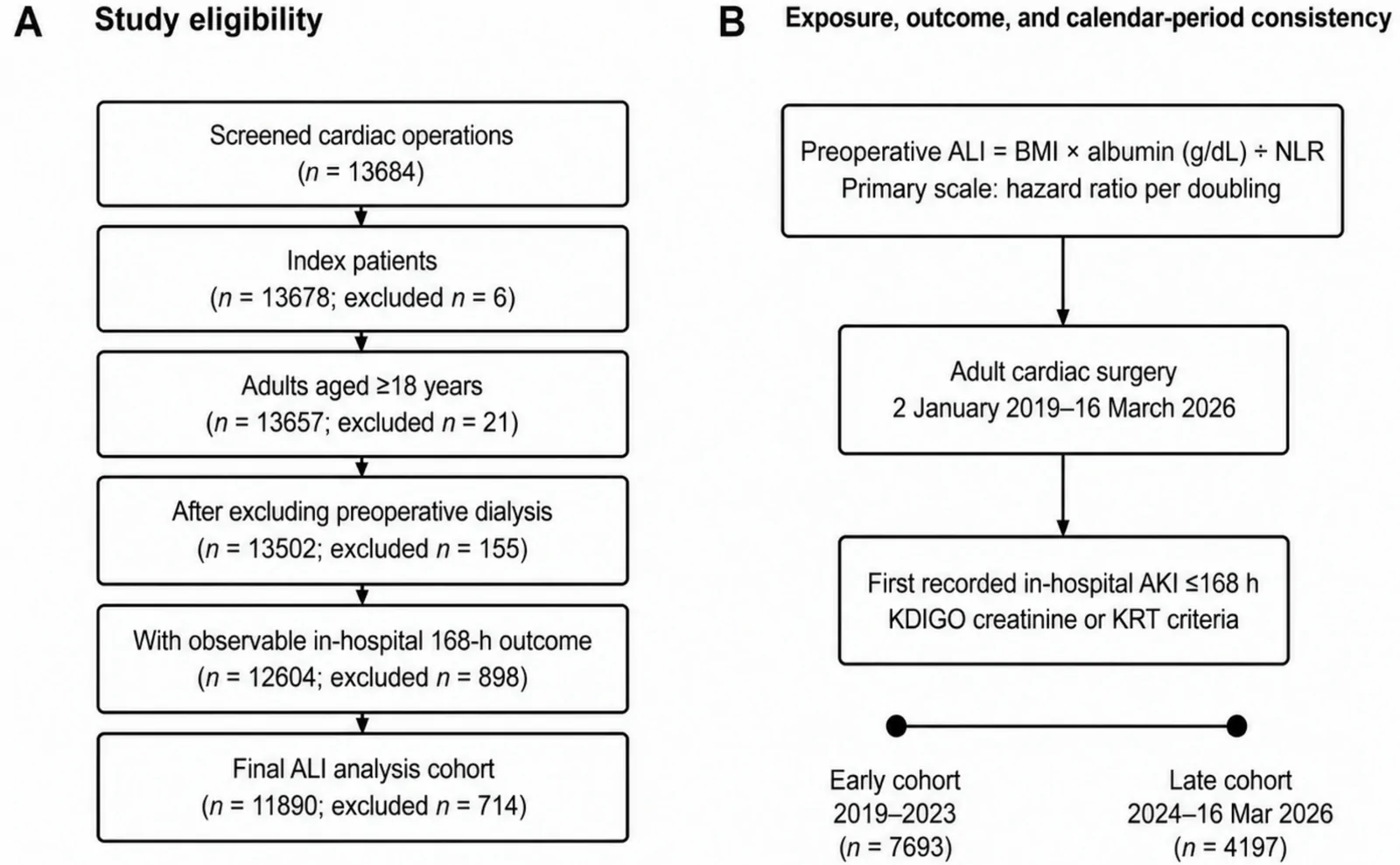
Study eligibility, exposure definition, outcome observation window, and calendar-period consistency cohorts. Panel (**A**) shows participant flow from 13,684 screened cardiac operations to 11,890 adults. Panel (**B**) defines preoperative ALI, the first in-hospital recorded AKI within 168 h, and the two same-center calendar periods. AKI, acute kidney injury; ALI, advanced lung cancer inflammation index; BMI, body mass index; KDIGO, Kidney Disease: Improving Global Outcomes; NLR, neutrophil-to-lymphocyte ratio.

Among 13,684 cardiac surgery operations screened, six repeat operations were removed, leaving 13,678 index patients. After the prespecified exclusions, 11,890 adults remained (Figure 1). AKI was recorded in 3380 patients (28.4%): maximum stage 1 in 2634, stage 2 in 451, and stage 3 in 295. Eighty-one patients received postoperative kidney replacement therapy within 168 h; KRT was the first qualifying event source in 11, whereas 3369 first events were identified by creatinine. AKI was detected within 0–6 h in 568 patients. Timed laboratory-system creatinine supplied baseline values for 11,831 patients and the fallback field for 59; the median timed-baseline look-back was 100.2 h [55.7–147.6].

First-day creatinine coverage was uniformly high (99.4–99.5% across ALI quartiles; adjusted *p* = 0.963), but later nonoverlapping windows showed differential monitoring. Among patients at risk at interval start, measurement coverage was 76.0% in Q1 versus 70.3% in Q4 during >24–48 h, 55.5% versus 49.0% during >48–72 h, and 78.1% versus 69.8% during >72–168 h; adjusted *p* values were <0.001 for all three comparisons. The same pattern remained among patients observed and event-free through interval end. These findings support high early testing but not equivalent surveillance throughout the 168 h period (Tables S10 and S14).

Median preoperative ALI was 42.3 [28.8–59.2]. Patients with recorded AKI had lower ALI than those without recorded AKI (37.2 [24.4–54.1] vs. 44.3 [30.6–60.6]). Recalculated eGFR was complete and had a median of 94.7 [79.2–103.1] mL/min/1.73 m^2^; 82 paired records changed classification at the 60 mL/min/1.73 m^2^ threshold relative to the stored field. Documented heart failure comprised 8687 patients (73.1%). Among 6484 patients with heart failure and available LVEF, 5025 (77.5%) had LVEF ≥ 50%, confirming that the diagnosis was not restricted to reduced-LVEF heart failure (Table 1, Tables S6 and S15).

### 3.2. Primary Association and Dose–Response

**Figure 2:**
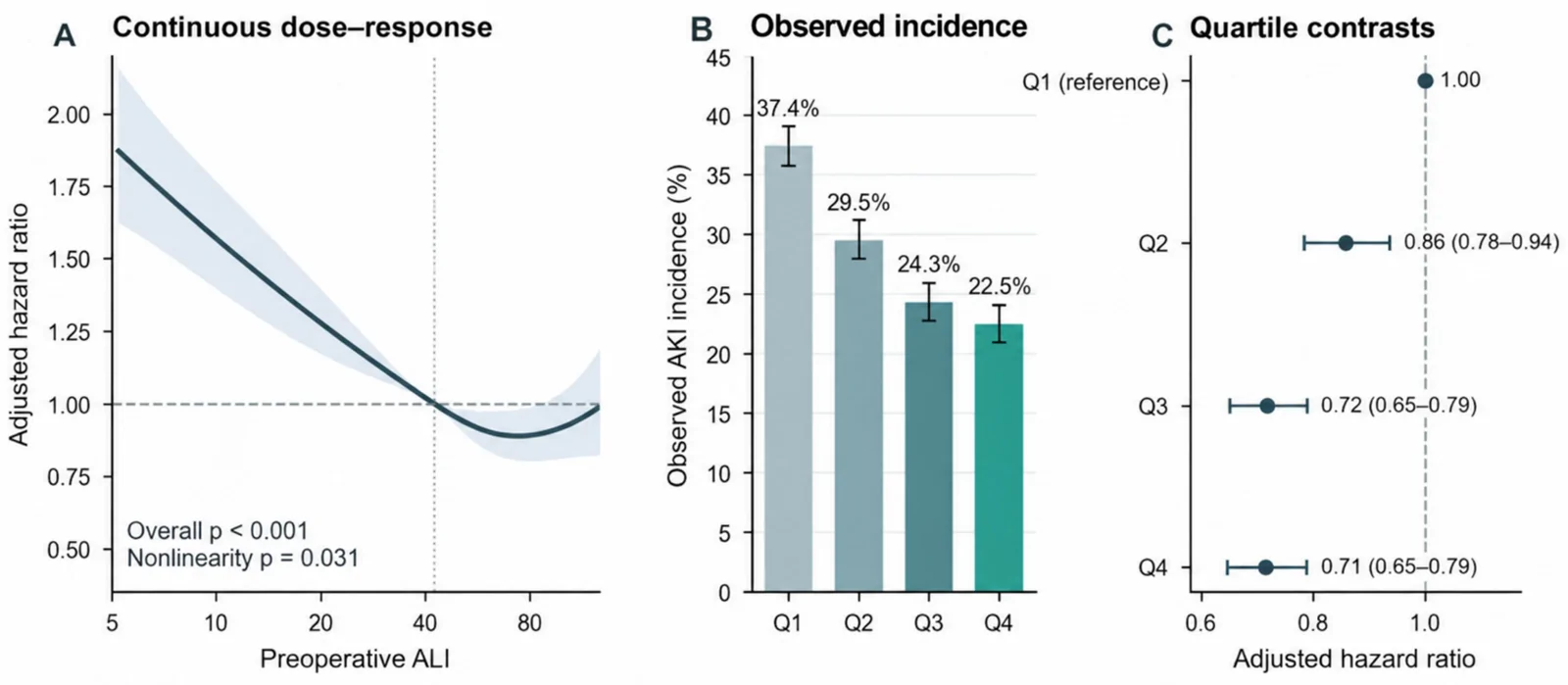
Continuous and categorical associations between preoperative ALI and postoperative AKI. Panel (**A**) shows the adjusted restricted cubic spline relative to the median ALI (42.3), with 95% CI; the ALI axis is displayed on a base-2 logarithmic scale while tick labels remain in native ALI units. The dashed horizontal line denotes HR = 1 and the dotted vertical line the reference. Panel (**B**) shows observed AKI incidence by ALI quartile with Wilson 95% CIs. Panel (**C**) shows M2-adjusted quartile HRs using quartile 1 as the reference. AKI, acute kidney injury; ALI, advanced lung cancer inflammation index; CI, confidence interval; HR, hazard ratio.

Each doubling of ALI was associated with a lower recorded AKI rate in M0 (HR, 0.75; 95% CI, 0.72–0.77), M1 (HR, 0.77; 95% CI, 0.74–0.80), and M2 (HR, 0.83; 95% CI, 0.80–0.86); all *p* < 0.001. The supportive modified Poisson RR was 0.86 (95% CI, 0.83–0.88) and remained 0.86 (95% CI, 0.84–0.89) after excluding the 600 shorter observations (Table S11). Standard scaled-Schoenfeld-residual testing indicated nonproportionality for ALI (χ^2^ = 6.71; *p* = 0.010) and globally (χ^2^ = 313.04; df = 12; *p* < 0.001). Interval-specific HRs were 0.87 (95% CI, 0.83–0.90) for 0–24 h, 0.72 (95% CI, 0.67–0.77) for >24–72 h, and 0.87 (95% CI, 0.72–1.04; *p* = 0.129) for >72–168 h. The M2 HR was therefore interpreted as an average cause-specific association over the observed postoperative period (Table 2, Figure 2 and Figure S2).

The spline model supported an overall association between ALI and AKI (*p* < 0.001), with modest evidence of nonlinearity (*p* = 0.031). Observed AKI incidence decreased across ALI quartiles. Compared with quartile 1, adjusted HRs were 0.86 (95% CI, 0.78–0.94; *p* < 0.001) for quartile 2, 0.72 (95% CI, 0.65–0.79; *p* < 0.001) for quartile 3, and 0.71 (95% CI, 0.65–0.79; *p* < 0.001) for quartile 4.

### 3.3. Component Decomposition

**Figure 3:**
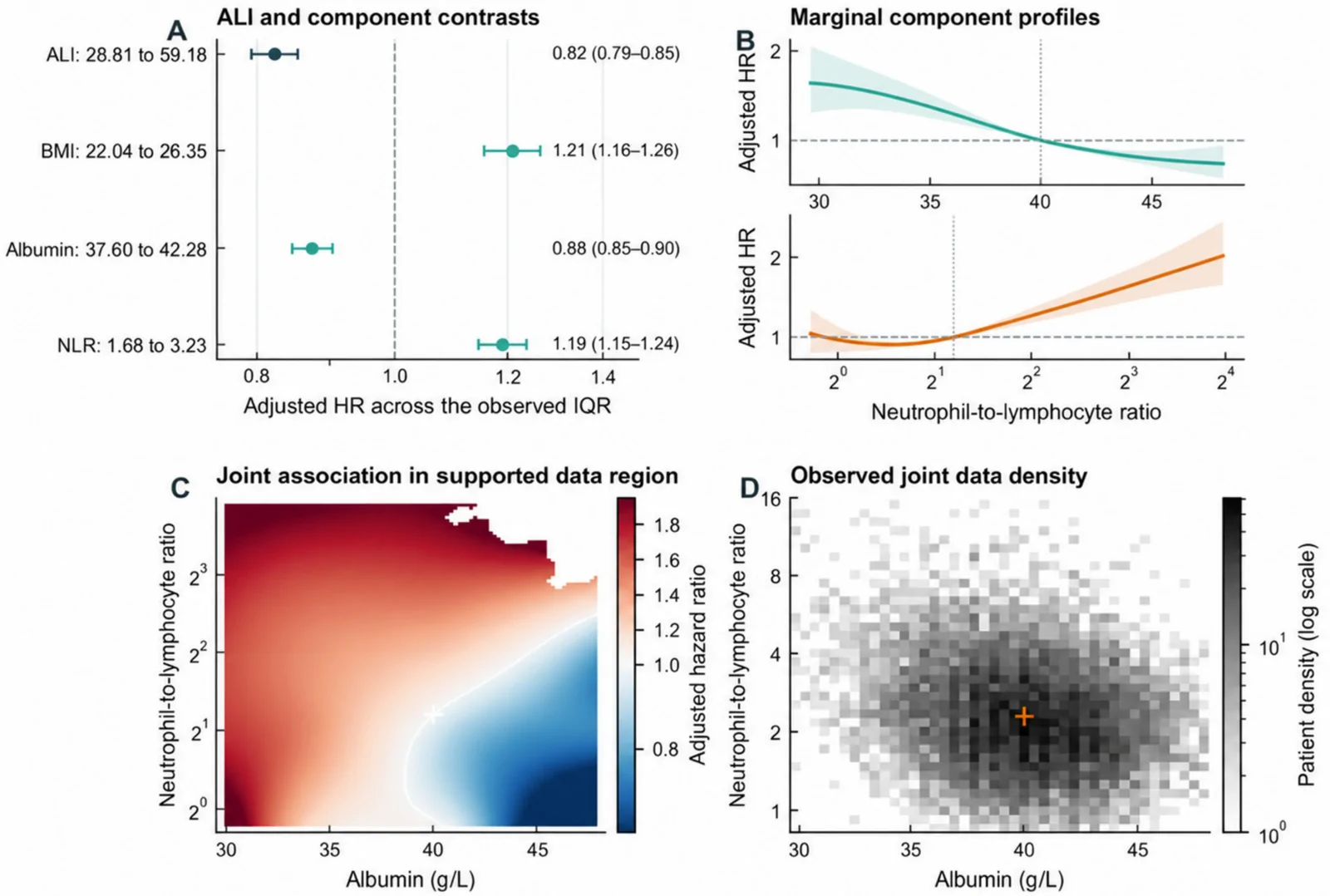
ALI component associations and the joint albumin–NLR pattern. Panel (**A**) reports adjusted HRs across observed interquartile-range contrasts for ALI and its components; ALI was modeled with M2 covariates, whereas BMI, albumin, and NLR were entered jointly. Dark blue denotes ALI, and teal denotes the individual components. The vertical dashed line indicates HR = 1. Panel (**B**) shows adjusted marginal spline profiles for albumin (teal) and NLR (orange), holding the other component at its median; BMI is summarized in Panel (**A**) because the flexible joint analysis was designed to examine the albumin–NLR ratio structure. Horizontal dashed lines indicate HR = 1, and vertical dotted lines indicate the median value of the corresponding component. Panel (**C**) shows the adjusted joint HR surface relative to median albumin and NLR; sparsely supported combinations are masked. Colors represent the adjusted HR, with red indicating higher HRs, blue indicating lower HRs, and white corresponding approximately to HR = 1. The white star marks the reference combination of median albumin and median NLR. Panel (**D**) displays the observed joint patient density; darker shading indicates greater patient density, and the orange plus sign marks the median albumin–NLR reference combination. The joint surface is exploratory and describes adjusted association rather than causal interaction. AKI, acute kidney injury; ALI, advanced lung cancer inflammation index; BMI, body mass index; CI, confidence interval; HR, hazard ratio; NLR, neutrophil-to-lymphocyte ratio.

In the mutually adjusted component model, IQR increases in BMI, albumin, and NLR had HRs of 1.21 (95% CI, 1.16–1.26), 0.88 (95% CI, 0.85–0.90), and 1.19 (95% CI, 1.15–1.24), respectively. Flexible BMI modeling showed nonlinearity (LR χ^2^ = 22.46; df = 3; *p* < 0.001), but the IQR contrast remained positive (HR, 1.21; 95% CI, 1.12–1.30). The free BMI–albumin–NLR model fit better than constrained ALI (LR χ^2^ = 135.68; df = 2; *p* < 0.001). The albumin–NLR tensor interaction was also detectable (LR χ^2^ = 35.62; df = 9; *p* < 0.001); the joint low-albumin/high-NLR versus high-albumin/low-NLR IQR contrast had an HR of 1.58 (95% CI, 1.44–1.74). These are association contrasts, not causal interaction or prediction performance results (Table 3, Tables S4, S9 and S17, and Figure 3).

### 3.4. Secondary, Calendar Period, and Sensitivity Analyses

Mutually exclusive classification identified 3771 isolated CABG, 4551 isolated valve, 329 isolated aortic, 2720 combined major-domain, and 519 other operations. Stratum HRs ranged from 0.69 in isolated aortic surgery to 0.91 in isolated valve surgery. Although the combined-versus-noncombined interaction was *p* = 0.079, the global seven-degree-of-freedom interaction across all mutually exclusive categories was significant (LR χ^2^ = 43.93; *p* < 0.001), indicating heterogeneity in association magnitude. Of the 519 other operations, 19 involved heart transplantation, five had a congenital surgery indicator, and 495 had none of the five named procedure indicators. Operative models moved the HR toward 1.00, reaching 0.90 (95% CI, 0.87–0.94) in the full mechanical support model. The post hoc model adding six available preoperative clinical state indicators produced the same rounded HR of 0.90 (95% CI, 0.87–0.94; *p* < 0.001), showing attenuation rather than complete confounding control (Table 4, Tables S2, S3 and S16).

Selection weighting improved measured covariate balance: after weighting, absolute SMDs were below 0.03 for all available modeled covariates. The baseline-creatinine-missingness indicator remained imbalanced (absolute SMD, 0.364) because no selected patient lacked a valid baseline value. The effective sample size was 11,776, and the weighted HR was 0.81 (95% CI, 0.78–0.84; *p* < 0.001). Weighting therefore addressed measured distributional differences but could not reconstruct outcomes for patients missing the information required for ascertainment (Tables S7, S8 and S18).

**Figure 4:**
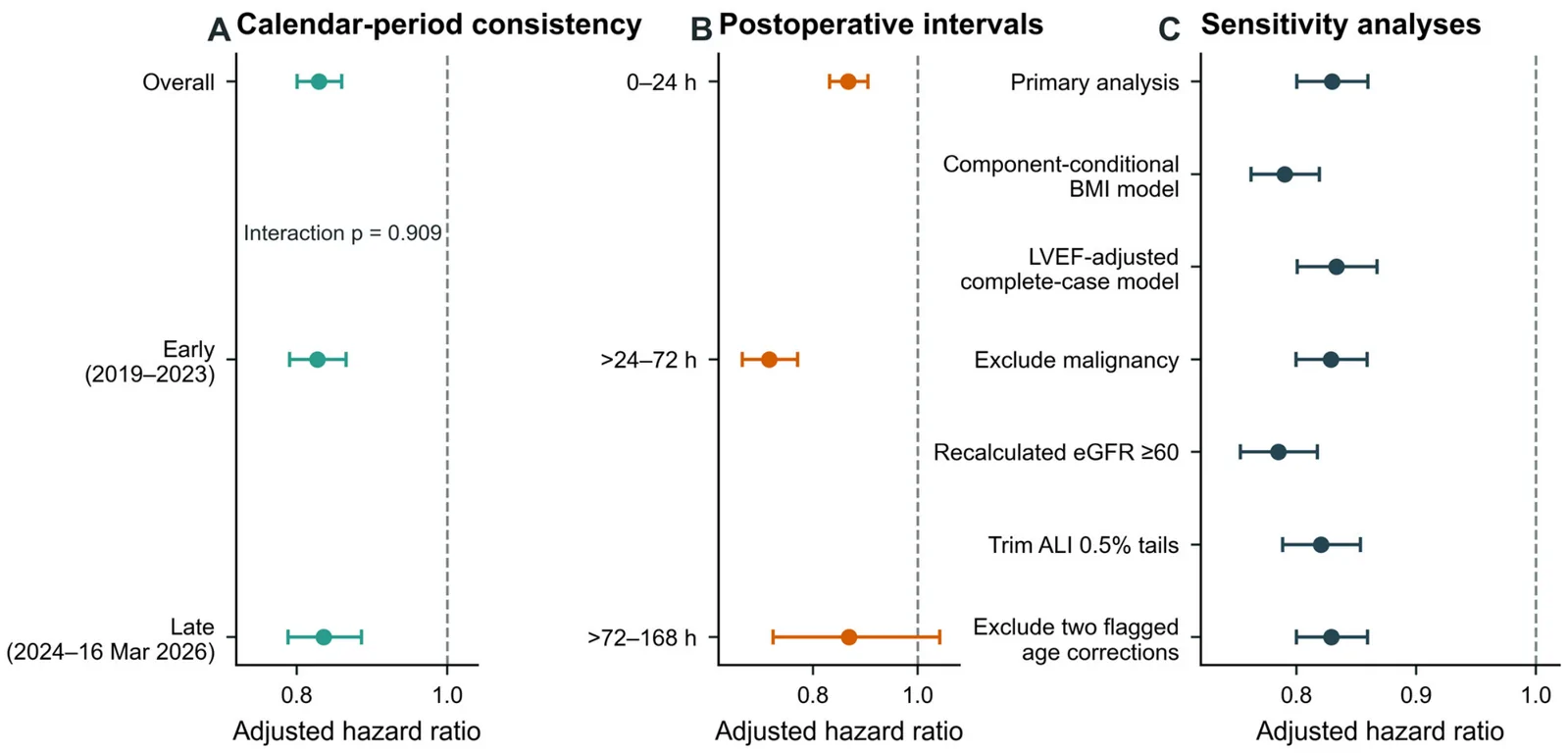
Calendar period consistency, postoperative interval-specific estimates, and sensitivity analyses for the association between preoperative ALI and postoperative AKI. Panel (**A**) shows the overall, early-period (2019–2023), and late-period (1 January 2024–16 March 2026) M2-adjusted HRs per doubling of ALI; the early-versus-late interaction *p* value was 0.909. Panel (**B**) shows post hoc cause-specific Cox estimates for 0–24, >24–72, and >72–168 h among patients event-free and under observation at each interval start. Panel (**C**) shows sensitivity analyses, including the component-conditional BMI model, the LVEF-adjusted complete-case model, and exclusion of two adult records flagged for large corrected-age differences. Points denote adjusted HRs and horizontal lines 95% CIs; dashed vertical lines denote HR = 1. AKI, acute kidney injury; ALI, advanced lung cancer inflammation index; BMI, body mass index; CI, confidence interval; eGFR, estimated glomerular filtration rate; HR, hazard ratio; LVEF, left ventricular ejection fraction.

Omitting heart failure left the primary estimate unchanged (HR, 0.83; 95% CI, 0.80–0.86). Within the same 8995 LVEF-complete patients, the M2 HR was 0.82 (95% CI, 0.79–0.86) before and 0.83 (95% CI, 0.80–0.87) after adding LVEF. Recalculated eGFR ≥60 defined 10,728 patients with 2739 events and yielded an HR of 0.78 (95% CI, 0.75–0.82). The supportive severe-AKI OR was 0.67 (95% CI, 0.63–0.73) and remained 0.67 (95% CI, 0.62–0.73) after excluding 718 shorter observations. Same-center calendar-period estimates remained similar (interaction *p* = 0.909) (Table 4, Tables S12 and S15; Figure 4).

## 4. Discussion

In this cohort of 11,890 adults, higher preoperative ALI was associated with a lower rate of in-hospital AKI recorded within 168 h. The association was not uniform: it attenuated from HR 0.83 to approximately 0.90 after adjustment for available operative or preoperative clinical state variables, varied across procedure categories, and was accompanied by differential creatinine monitoring after the first postoperative day. The most robust interpretative result was that the fixed component relationship encoded by ALI fit less well than freely estimated BMI, albumin, and NLR effects.

Earlier cardiac surgery studies have linked preoperative or early postoperative blood cell indices with AKI, but they differ in population, predictor timing, and analytical purpose [8,9,10,11,12,13]. Some studies evaluated association, whereas others used outcome-derived cutoffs or reported discrimination from small cohorts. Nutritional indices, albumin-containing ratios, fibrinogen, and iron metabolism markers have also been studied in relation to AKI, and a recent two-center study combined nutritional indices with machine-learning prediction [14,15,16,17,18]. The present analysis addresses a narrower question: whether a preoperative composite of routinely available inflammatory and nutritional measurements is associated with recorded postoperative AKI across a broad adult cardiac-surgery population. It does not evaluate whether ALI improves an established clinical risk model.

ALI was developed in advanced lung cancer, but its components are not tumor-specific. Albumin, BMI, and NLR reflect aspects of nutritional reserve, systemic inflammation, and immune balance that are also relevant to the inflammatory, ischemic, and hemodynamic stress of cardiac surgery [1,4,5,6,7]. This biological overlap supports studying the index in cardiac surgery cohorts, although the observed component discordance argues against transferring its oncologic interpretation without re-evaluation.

The component findings limit interpretation of ALI as a unified construct. Albumin and NLR followed the expected directions, whereas BMI remained positively associated with recorded AKI even after flexible modeling. ALI forces the BMI and albumin coefficients to have the same magnitude and the NLR coefficient to have the opposite magnitude; the formal test rejected these constraints. The albumin–NLR surface further showed that joint component patterns were not well summarized by a fixed ratio. These findings concern association structure, not predictive superiority or causality.

The same-center calendar comparison showed temporal consistency but not external validity. Standard PH testing and the piecewise estimates confirmed that a single HR averages a changing association. The fixed-horizon RR was directionally similar, but its full-cohort version treated all unrecorded outcomes as non-events. Moreover, surveillance after 24 h was more frequent in the lowest ALI quartile. The data therefore support an association with recorded inpatient AKI, not complete seven-day risk independent of observation.

ALI can be calculated from routine preoperative measurements without an additional specimen. The central contribution of this analysis is therefore not only the inverse ALI–AKI association, but also the demonstration that the equal-weight component structure built into ALI was not supported in this cardiac surgery cohort. ALI may remain a parsimonious summary marker, but future multicenter studies should compare it directly with freely modeled components and established renal risk models using external discrimination, calibration, and decision-analytic measures.

Several measurement limitations remain. The locked extract retained the selected baseline, outcome flags, first-event trace, and maximum stage but not the complete creatinine-value trajectory or upstream derivation program. Although the source pipeline was specified to use KDIGO creatinine and KRT criteria, every pairwise comparison could not be rerun from the distributed files. Urine output and a separate preoperative-AKI phenotype were unavailable. ALI components were stored in preoperative fields without a common timestamp, so exact look-back and same-day concordance could not be verified.

Outcome observation was clinically driven. Early creatinine testing was nearly universal, but monitoring differed in later intervals and could both affect detection and respond to evolving illness. Discharge and death ended inpatient observation; excluding shorter observations changed the analysis population and did not recover unobserved post-discharge AKI. The full-cohort Poisson and severe-AKI models are therefore supportive analyses of recorded outcomes rather than complete 168 h cumulative incidence.

Residual confounding is also likely. Adjustment for available preoperative clinical-state indicators and perioperative variables attenuated the association, but the latter may include downstream factors. Procedure-specific magnitudes differed, documented heart failure lacked source-level validation, and LVEF was incomplete. Selection weighting improved measured balance but could not correct baseline-creatinine missingness or unmeasured selection. Finally, the single-center design and same-center calendar comparison limit transportability.

## 5. Conclusions

Lower preoperative ALI was associated with a higher rate of in-hospital AKI recorded after adult cardiac surgery. However, later differential monitoring, attenuation after clinical state adjustment, time-varying effects, and procedure heterogeneity limit a uniform interpretation. The discordant and nonlinear BMI association and rejection of ALI’s fixed component constraints indicate that the composite formula requires re-evaluation. External studies should validate the outcome algorithm and compare ALI directly with flexibly modeled components and established clinical models.

## Figures and Tables

**Table 1:** Baseline characteristics overall and by postoperative AKI status.

Characteristic	Overall (*N* = 11,890)	No AKI (*n* = 8510)	AKI (*n* = 3380)	SMD
Age, years	61.7 (11.5)	60.8 (11.6)	63.9 (10.7)	0.27
Female sex	4459 (37.5%)	3366 (39.6%)	1093 (32.3%)	−0.15
BMI, kg/m^2^	24.3 (3.4)	24.1 (3.3)	24.7 (3.7)	0.17
Albumin, g/L	40.0 [37.6–42.3]	40.3 [38.0–42.5]	39.1 [36.6–41.5]	−0.34
Neutrophils, ×10^9^/L	3.8 [2.9–4.8]	3.7 [2.9–4.7]	4.0 [3.0–5.2]	0.27
Lymphocytes, ×10^9^/L	1.6 [1.2–2.0]	1.6 [1.3–2.1]	1.5 [1.2–1.9]	−0.21
NLR	2.3 [1.7–3.2]	2.2 [1.6–3.1]	2.5 [1.8–3.8]	0.28
ALI	42.3 [28.8–59.2]	44.3 [30.6–60.6]	37.2 [24.4–54.1]	−0.26
Baseline creatinine, μmol/L	72.5 [61.0–86.7]	70.2 [59.5–82.4]	80.0 [65.8–99.0]	0.36
Baseline eGFR, mL/min/1.73 m^2^	94.7 [79.2–103.1]	96.6 [84.9–104.1]	87.3 [66.6–98.9]	−0.53
LVEF, %	58.0 (9.8)	58.8 (9.2)	55.9 (11.0)	−0.28
Diabetes	3247 (27.3%)	2132 (25.1%)	1115 (33.0%)	0.18
Hypertension	6213 (52.3%)	4143 (48.7%)	2070 (61.2%)	0.25
Documented preoperative heart failure	8687 (73.1%)	6210 (73.0%)	2477 (73.3%)	0.01
CABG-containing surgery	5232 (44.0%)	3756 (44.1%)	1476 (43.7%)	−0.01
Valve-containing surgery	7230 (60.8%)	5002 (58.8%)	2228 (65.9%)	0.15
Aortic surgery	1844 (15.5%)	1202 (14.1%)	642 (19.0%)	0.13
Congenital surgery	60 (0.5%)	41 (0.5%)	19 (0.6%)	0.01
Heart transplantation	25 (0.2%)	9 (0.1%)	16 (0.5%)	0.07

Data are mean (standard deviation), median [interquartile range], or number/denominator (percentage). Documented preoperative heart failure was the union of the mutually exclusive structured chronic and acute source categories; the extract did not preserve the precise clinical record origin and the variable was not defined by an LVEF threshold. CABG-containing, valve-containing, and aortic surgery indicators are nonexclusive; percentages therefore do not sum to 100%. eGFR was recalculated for all 11,890 patients with the 2021 CKD-EPI creatinine equation; the available-case sample size for LVEF was 8995, and all other variables were complete. Signed SMDs were calculated as the AKI group minus the no-AKI group. AKI, acute kidney injury; ALI, advanced lung cancer inflammation index; BMI, body mass index; CABG, coronary artery bypass grafting; eGFR, estimated glomerular filtration rate; LVEF, left ventricular ejection fraction; NLR, neutrophil-to-lymphocyte ratio; SMD, standardized mean difference.

**Table 2:** Primary association, quartile contrasts, and dose–response tests.

Analysis	Contrast	Estimate (95% CI)	*p* Value
M0	ALI per doubling	0.75 (0.72–0.77)	<0.001
M1	ALI per doubling	0.77 (0.74–0.80)	<0.001
M2	ALI per doubling	0.83 (0.80–0.86)	<0.001
M2 quartiles	Q2 vs. Q1	0.86 (0.78–0.94)	<0.001
M2 quartiles	Q3 vs. Q1	0.72 (0.65–0.79)	<0.001
M2 quartiles	Q4 vs. Q1	0.71 (0.65–0.79)	<0.001
Spline	Overall association	LR chi-square = 107.53	<0.001
Spline	Nonlinearity	LR chi-square = 8.88	0.031

M0 included ALI alone. M1 added corrected age and sex. M2 additionally included log2 baseline creatinine, diabetes, hypertension, heart failure, and surgical domain indicators. Observed ALI ranges (n/events) were Q1, 0.94–28.81 (2973/1113); Q2, >28.81–42.29 (2972/877); Q3, >42.29–59.16 (2972/722); and Q4, >59.16–389.02 (2973/668). CI, confidence interval; HR, hazard ratio; LR, likelihood ratio.

**Table 3:** ALI and component contrasts, coefficient-constraint test, and severe AKI analysis.

Analysis	Observed Interquartile-Range Contrast	Adjusted Estimate (95% CI) or Test Statistic	*p* Value
ALI	28.81 to 59.18 ALI units	0.82 (0.79–0.85)	<0.001
BMI	22.04 to 26.35 kg/m^2^	1.21 (1.16–1.26)	<0.001
Albumin	37.60 to 42.28 g/L	0.88 (0.85–0.90)	<0.001
NLR	1.68 to 3.23	1.19 (1.15–1.24)	<0.001
Severe AKI	ALI per doubling	OR 0.67 (0.63–0.73)	<0.001
ALI coefficient constraint	Constrained ALI vs. free BMI–albumin–NLR coefficients	LR χ^2^ = 135.68 (df = 2)	<0.001

ALI was estimated in M2. BMI, albumin, and NLR were entered jointly with M2 covariates and re-expressed across their observed interquartile ranges. The severe AKI row reports the supportive full-cohort adjusted odds ratio; patients without documented stage 2 or 3 AKI were coded as non-events, including those with shorter observation. The coefficient constraint test compares the nested ALI model with the free BMI–albumin–NLR model on the same 11,890 patients and 3380 AKI events. AKI, acute kidney injury; ALI, advanced lung cancer inflammation index; BMI, body mass index; CI, confidence interval; HR, hazard ratio; NLR, neutrophil-to-lymphocyte ratio; OR, odds ratio.

**Table 4:** Calendar period, procedural, perioperative, and selection bias analyses for the association per doubling of ALI.

Analysis	N	AKI Events	Adjusted HR (95% CI)	*p* Value
Overall cohort	11,890	3380	0.83 (0.80–0.86)	<0.001
Early cohort (2019–2023)	7693	2149	0.83 (0.79–0.87)	<0.001
Late cohort (2024–16 Mar 2026)	4197	1231	0.84 (0.79–0.89)	<0.001
Early vs. late interaction	—	—	—	0.909
Component-conditional BMI model	11,890	3380	0.79 (0.76–0.82)	<0.001
M2 on LVEF-complete frame	8995	2591	0.82 (0.79–0.86)	<0.001
M2 plus LVEF on same frame	8995	2591	0.83 (0.80–0.87)	<0.001
M2 without heart failure	11,890	3380	0.83 (0.80–0.86)	<0.001
Exclude malignancy	11,667	3347	0.83 (0.80–0.86)	<0.001
Recalculated eGFR ≥60	10,728	2739	0.78 (0.75–0.82)	<0.001
Trim ALI 0.5% tails	11,770	3331	0.82 (0.79–0.85)	<0.001
Exclude flagged age corrections	11,888	3379	0.83 (0.80–0.86)	<0.001
Mutually exclusive procedure-category adjustment	11,890	3380	0.85 (0.82–0.89)	<0.001
Global ALI-by-procedure-category interaction	11,890	3380	LR χ^2^ = 43.93 (df = 7)	<0.001
Isolated CABG	3771	870	0.89 (0.81–0.97)	0.007
Isolated valve surgery	4551	1275	0.91 (0.86–0.97)	0.004
Isolated aortic surgery	329	166	0.69 (0.61–0.78)	<0.001
Combined major-domain surgery	2720	971	0.86 (0.81–0.92)	<0.001
CPB-duration operative model	11,890	3380	0.89 (0.86–0.92)	<0.001
Cross-clamp-duration operative model	11,890	3380	0.87 (0.84–0.90)	<0.001
Full operative and mechanical support model	11,890	3380	0.90 (0.87–0.94)	<0.001
Preoperative clinical state model	11,890	3380	0.90 (0.87–0.94)	<0.001
Selection probability weighted model	11,890	3380	0.81 (0.78–0.84)	<0.001

Early and late cohorts were defined by surgery date. Procedure-category analyses used mutually exclusive combinations; the global interaction compared all category-specific ALI slopes. The LVEF rows use the same complete-case frame before and after LVEF adjustment. eGFR was recalculated from corrected age, sex, and baseline creatinine. The preoperative clinical state model added cardiogenic shock, cardiopulmonary resuscitation, tracheal intubation, infective endocarditis, acute aortic dissection or intramural hematoma, and intravenous catecholamine use. The full operative model included both duration measures, IABP, and ECMO and was interpreted as downstream-inclusive. AKI, acute kidney injury; ALI, advanced lung cancer inflammation index; CABG, coronary artery bypass grafting; CI, confidence interval; CPB, cardiopulmonary bypass; ECMO, extracorporeal membrane oxygenation; HR, hazard ratio; IABP, intra-aortic balloon pump; LVEF, left ventricular ejection fraction.

## Data Availability

The de-identified analytic data are not publicly available because they contain institutionally governed patient-level clinical information. Requests may be considered by the corresponding author and Nanjing First Hospital, subject to ethics approval, data-use agreements, and applicable privacy requirements. Supplementary Code S1 provides reproducible analysis and cohort-eligibility code beginning from the locked outcome-coded extracts; it does not include patient-level data, the complete creatinine-value trajectory, or the upstream AKI-derivation program.

## Supplementary Materials

The following supporting information can be downloaded at https://www.mdpi.com/article/10.3390/jcdd13100490/s1, Table S1: Baseline characteristics by calendar-period cohort; Table S2: Sensitivity and perioperative-adjustment analyses; Table S3: Mutually exclusive procedure categories and procedure-stratified associations; Table S4: IQR-scaled ALI and component associations; Table S5: Interval-specific cause-specific Cox analyses; Table S6: Documented heart-failure categories and LVEF distribution; Table S7: Characteristics of included and excluded patients; Table S8: Outcome observability and selection-weight diagnostics; Table S9: Association-model fit for ALI and its components; Table S10: Cumulative postoperative creatinine-monitoring coverage by ALI quartile; Table S11: Fixed-horizon modified Poisson analyses for AKI within 168 h; Table S12: Severe-AKI follow-up sensitivity analysis; Table S13: Outcome construction and fixed-horizon coding audit; Table S14: Nonoverlapping postoperative creatinine-monitoring intervals by ALI quartile; Table S15: eGFR recalculation and same-frame sensitivity analyses; Table S16: Procedure heterogeneity, other-surgery composition, and preoperative clinical state; Table S17: Flexible component analyses and ALI spline knots; Table S18: Proportional-hazards and selection-weight diagnostics; Figure S1: IQR-scaled components and mutually exclusive surgery strata; Figure S2: Scaled Schoenfeld residuals for log2 ALI; Supplementary Code S1: Reproducible Python (versions 3.13.5 and 3.10.15) and R (version 4.5.2) analysis code.
