# Supplementary material for: Preoperative Advanced Lung Cancer Inflammation Index and Acute Kidney Injury After Adult Cardiac Surgery: A Retrospective Cohort Study
Source: J Cardiovasc Dev Dis. 2026 Sep 30;13(10):490. doi: 10.3390/jcdd13100490 (PMC13705152; doi:10.3390/jcdd13100490)
Supplement: Supplementary file 1 [file jcdd-13-00490-s001.zip › jcdd-4518666-Supplementary Material.pdf]

Supplementary Materials

Manuscript ID: jcdd-4518666

Preoperative Advanced Lung Cancer Inflammation Index and Acute Kidney Injury After Adult Cardiac Surgery: A Retrospective Cohort Study

Haoran Gan, Ruoyu Zhang, Rui Fan, Li Yin, Shenjie Ning, Junzhe Wang, Dongming Liu, Wen Chen, Xin Chen, and Zhibing Qiu

Table S1. Baseline characteristics by calendar-period cohort

Characteristic	Overall (N=11,890)	Early (2019–2023) (N=7,693)	Late (2024–16 March 2026) (N=4,197)	SMD
Age, years	61.7 (11.5)	61.2 (11.6)	62.6 (11.1)	0.128
Female sex	4459 (37.5%)	2958 (38.5%)	1501 (35.8%)	-0.056
BMI, kg/m²	24.3 (3.4)	24.2 (3.4)	24.5 (3.4)	0.098
Albumin, g/L	40.0 [37.6-42.3]	40.1 [37.7-42.4]	39.8 [37.4-42.0]	-0.093
Neutrophils, 10^9/L	3.8 [2.9-4.8]	3.8 [2.9-4.8]	3.8 [2.9-4.8]	0.020
Lymphocytes, 10^9/L	1.6 [1.2-2.0]	1.6 [1.3-2.0]	1.6 [1.2-2.0]	-0.057
NLR	2.3 [1.7-3.2]	2.3 [1.7-3.2]	2.3 [1.7-3.3]	0.026
ALI	42.3 [28.8-59.2]	42.5 [29.2-59.4]	41.8 [28.1-58.6]	-0.029
Baseline creatinine, μmol/L	72.5 [61.0-86.7]	72.9 [61.0-87.0]	72.2 [60.8-86.1]	0.011
Baseline eGFR, mL/min/1.73 m²	94.7 [79.2–103.1]	94.9 [79.0–103.4]	94.5 [79.9–102.5]	−0.024
LVEF, %	58.0 (9.8)	58.2 (9.6)	57.5 (10.3)	-0.068
Diabetes	3247 (27.3%)	1992 (25.9%)	1255 (29.9%)	0.089
Hypertension	6213 (52.3%)	4017 (52.2%)	2196 (52.3%)	0.002
Documented preoperative heart failure	8687 (73.1%)	4953 (64.4%)	3734 (89.0%)	0.608
CABG-containing surgery	5232 (44.0%)	3278 (42.6%)	1954 (46.6%)	0.079
Valve-containing surgery	7230 (60.8%)	4718 (61.3%)	2512 (59.9%)	-0.030
Aortic surgery	1844 (15.5%)	1122 (14.6%)	722 (17.2%)	0.072
Congenital surgery	60 (0.5%)	42 (0.5%)	18 (0.4%)	-0.017

Data are mean (standard deviation), median [interquartile range], or n (%). Calendar periods are defined by surgery date. SMDs are signed differences for the late minus early cohort. Heart failure was source-recorded and was not defined by LVEF. Surgery-domain indicators are nonexclusive. LVEF summaries use available measurements. BMI, body mass index; eGFR, estimated glomerular filtration rate; LVEF, left ventricular ejection fraction; SMD, standardized mean difference.

Table S2. Sensitivity and perioperative-adjustment analyses

Analysis	N	AKI events	Adjusted HR (95% CI)	p value
Primary analysis	11,890	3,380	0.83 (0.80–0.86)	<0.001
Component-conditional BMI model	11,890	3,380	0.79 (0.76–0.82)	<0.001
M2 on LVEF-complete frame	8,995	2,591	0.82 (0.79–0.86)	<0.001
M2 plus LVEF on same frame	8,995	2,591	0.83 (0.80–0.87)	<0.001
M2 without documented heart failure	11,890	3,380	0.83 (0.80–0.86)	<0.001
Exclude malignancy	11,667	3,347	0.83 (0.80–0.86)	<0.001
Recalculated eGFR ≥60	10,728	2,739	0.78 (0.75–0.82)	<0.001
Trim ALI 0.5% tails	11,770	3,331	0.82 (0.79–0.85)	<0.001
Exclude flagged age corrections	11,888	3,379	0.83 (0.80–0.86)	<0.001
Mutually exclusive procedure-category model	11,890	3,380	0.85 (0.82–0.89)	<0.001
Operative model with CPB duration	11,890	3,380	0.89 (0.86–0.92)	<0.001
Operative model with cross-clamp duration	11,890	3,380	0.87 (0.84–0.90)	<0.001
Full operative-duration model	11,890	3,380	0.90 (0.87–0.93)	<0.001
Full operative and mechanical-support model	11,890	3,380	0.90 (0.87–0.94)	<0.001
Preoperative clinical-state model	11,890	3,380	0.90 (0.87–0.94)	<0.001
Selection-probability-weighted model	11,890	3,380	0.81 (0.78–0.84)	<0.001

HRs are per ALI doubling from cause-specific Cox models. M2 adjusted for corrected age, sex, log2 baseline creatinine, diabetes, hypertension, documented heart failure, and CABG, valve, aortic, congenital-surgery, and transplantation indicators. The component-conditional BMI model additionally included log2 BMI. The LVEF rows use the same complete-case sample. Procedure-category and operative models replaced CABG, valve, and aortic indicators with mutually exclusive categories, retaining congenital and transplant indicators. Operative models also included sternotomy, repeat cross-clamping, distal venous anastomosis count, and valve-position count. Duration and mechanical-support additions are specified by row; CPB and cross-clamp durations were first fitted separately because of collinearity. These are downstream-inclusive sensitivity analyses. Exclusion and trimming rules follow main Methods. eGFR was recalculated from corrected age, sex, and baseline creatinine. Clinical-state covariates are defined in Table S16; selection weights in Tables S8 and S18. AKI, acute kidney injury; ALI, advanced lung cancer inflammation index; BMI, body mass index; CABG, coronary artery bypass grafting; CI, confidence interval; CPB, cardiopulmonary bypass; ECMO, extracorporeal membrane oxygenation; eGFR, estimated glomerular filtration rate; HR, hazard ratio; IABP, intra-aortic balloon pump; LVEF, left ventricular ejection fraction.

Table S3. Mutually exclusive procedure categories and procedure-stratified associations

Procedure category or analysis	N (%)	AKI events (%)	Adjusted HR (95% CI)	p value
Other cardiac surgery	519 (4.4%)	98 (18.9%)	-	-
Isolated CABG	3,771 (31.7%)	870 (23.1%)	0.89 (0.81-0.97)	0.007
Isolated valve surgery	4,551 (38.3%)	1,275 (28.0%)	0.91 (0.86-0.97)	0.004
Isolated aortic surgery	329 (2.8%)	166 (50.5%)	0.69 (0.61-0.78)	<0.001
CABG + valve surgery	1,205 (10.1%)	495 (41.1%)	-	-
CABG + aortic surgery	41 (0.3%)	18 (43.9%)	-	-
Valve + aortic surgery	1,259 (10.6%)	365 (29.0%)	-	-
CABG + valve + aortic surgery	215 (1.8%)	93 (43.3%)	-	-
Combined major-domain surgery (aggregate stratum)	2,720	971	0.86 (0.81-0.92)	<0.001
Other cardiac surgery (aggregate stratum)	519	98	0.71 (0.58-0.88)	0.002
ALI x combined-procedure interaction	11,890	3,380	Interaction ratio 1.07 (0.99-1.15)	0.079

The first eight rows partition the cohort by the CABG, valve, and aortic indicators. Their N percentages use the full cohort denominator; AKI percentages use each row's N. "Isolated" denotes only one of these three indicators being positive and does not exclude procedures outside those domains. Subsequent aggregate strata and interaction rows reuse patients and must not be added to the first eight rows. Stratum-specific models used the M2 nonprocedure covariates, congenital and transplant indicators, and any varying procedure-category indicators. HRs are per ALI doubling. The binary interaction compares combined with noncombined procedures; the global category interaction is reported in Table S16. AKI, acute kidney injury; ALI, advanced lung cancer inflammation index; CABG, coronary artery bypass grafting; CI, confidence interval; HR, hazard ratio.

Table S4. IQR-scaled ALI and component associations

Marker	IQR contrast	Adjusted HR (95% CI)	p value
ALI	28.81 to 59.18	0.82 (0.79-0.85)	<0.001
BMI	22.04 to 26.35	1.21 (1.16-1.26)	<0.001
Albumin	37.60 to 42.28	0.88 (0.85-0.90)	<0.001
NLR	1.68 to 3.23	1.19 (1.15-1.24)	<0.001

ALI was modeled separately with M2 covariates. BMI, albumin, and NLR were entered jointly with M2 covariates. Coefficients from log2 models were re-expressed across each marker's observed IQR. ALI, advanced lung cancer inflammation index; BMI, body mass index; CI, confidence interval; HR, hazard ratio; IQR, interquartile range; NLR, neutrophil-to-lymphocyte ratio.

Table S5. Interval-specific cause-specific Cox analyses

Postoperative interval	N at interval start	AKI events	Adjusted HR (95% CI)	p value
0-24 h	11,890	2,442	0.87 (0.83–0.90)	<0.001
>24-72 h	9,430	766	0.72 (0.67–0.77)	<0.001
>72-168 h	8,648	172	0.87 (0.72–1.04)	0.129

Time is measured from the documented end of surgery. Models were fitted among patients event-free and under observation at each interval start using the M2 adjustment set; HRs are per ALI doubling. The congenital-surgery indicator was omitted from the >72–168 h model because no AKI occurred among congenital-surgery patients in that interval. These post hoc interval analyses followed the formal scaled Schoenfeld-residual tests, which rejected proportional hazards for ALI and for the global model (Table S18 and Figure S2). AKI, acute kidney injury; ALI, advanced lung cancer inflammation index; CI, confidence interval; HR, hazard ratio.

Table S6. Documented heart-failure categories and LVEF distribution

Source-recorded category	N (% cohort)	LVEF available	LVEF, mean (SD)	LVEF <50%, n	LVEF ≥50%, n (% measured)
No documented heart failure	3,203 (26.9%)	2,511	60.8 (6.6)	255	2,256 (89.8%)
Chronic heart failure	7,676 (64.6%)	5,673	56.8 (10.8)	1,289	4,384 (77.3%)
Acute heart failure	1,011 (8.5%)	811	57.5 (9.5)	170	641 (79.0%)
Any documented heart failure	8,687 (73.1%)	6,484	56.9 (10.7)	1,459	5,025 (77.5%)

The source heart-failure variable was exactly the union of mutually exclusive chronic and acute categories. This comparison verified source-field coding consistency and did not independently adjudicate the clinical diagnoses. LVEF bands describe available measurements and did not define heart failure. LVEF, left ventricular ejection fraction; SD, standard deviation.

Table S7. Characteristics of included and excluded patients

Characteristic	Included (n=11,890)	Unobservable outcome (n=898)	SMD	Invalid ALI component (n=714)	SMD
Age, years	61.7 (11.5)	62.7 (11.2)	0.09	57.3 (13.2)	-0.35
Female sex	4459/11890 (37.5%)	329/898 (36.6%)	-0.02	212/714 (29.7%)	-0.17
BMI, kg/m2	24.3 (3.4)	24.2 (3.5)	-0.02	25.4 (4.0)	0.28
Baseline creatinine, umol/L	79.9 (46.3)	100.2 (97.3)	0.27	90.3 (70.7)	0.17
LVEF, %	58.0 (9.8)	58.6 (9.4)	0.07	61.0 (7.3)	0.35
Diabetes	3247/11890 (27.3%)	264/898 (29.4%)	0.05	116/714 (16.2%)	-0.27
Hypertension	6213/11890 (52.3%)	505/898 (56.2%)	0.08	481/714 (67.4%)	0.31
Documented heart failure	8687/11890 (73.1%)	803/898 (89.4%)	0.43	325/714 (45.5%)	-0.58
CABG-containing surgery	5232/11890 (44.0%)	401/898 (44.7%)	0.01	173/714 (24.2%)	-0.43
Valve-containing surgery	7230/11890 (60.8%)	514/898 (57.2%)	-0.07	330/714 (46.2%)	-0.30
Aortic surgery	1844/11890 (15.5%)	156/898 (17.4%)	0.05	430/714 (60.2%)	1.04

Continuous variables are mean (standard deviation); binary variables are n/available n (%). Exclusions are sequential: the invalid-ALI group had an observable outcome but a missing or nonpositive ALI component. SMDs are signed differences for each excluded group minus the included group. Available-case denominators can differ across variables. ALI, advanced lung cancer inflammation index; BMI, body mass index; CABG, coronary artery bypass grafting; LVEF, left ventricular ejection fraction; SMD, standardized mean difference.

Table S8. Outcome observability and selection-weight diagnostics

Mutually exclusive source outcome status	N	% of 898
No linked raw laboratory information	814	90.6%
No postoperative creatinine or kidney replacement therapy record	73	8.1%
Missing or invalid baseline creatinine	11	1.2%

The outcome-status rows are mutually exclusive reasons among the 898 patients with unobservable outcomes. Selection probabilities were estimated among 13,502 adult nondialysis index patients; missing predictors were median-imputed with missingness indicators and probabilities bounded at 0.01–0.99. Stabilized selection weights were truncated at the selected cohort's 1st and 99th percentiles (range, 0.890–1.516; effective sample size, 11,776.1). Weighted Cox inference used robust standard errors. Covariate balance and the residual baseline-creatinine missingness imbalance are shown in Table S18.

Analysis	N	AKI events	Adjusted HR (95% CI)	p value
Unweighted complete-cohort model	11,890	3,380	0.83 (0.80-0.86)	<0.001
Selection-probability-weighted model	11,890	3,380	0.81 (0.78-0.84)	<0.001

Table S9. Association-model fit for ALI and its components

Model	Parameters	Log likelihood	AIC	LR vs clinical model	p value
Clinical covariates only	11	-30686.1	61394.2	Reference	-
Clinical covariates + ALI	12	-30636.8	61297.5	98.6 (1 df)	<0.001
Clinical covariates + BMI	12	-30658.3	61340.6	55.6 (1 df)	<0.001
Clinical covariates + albumin	12	-30640.0	61304.1	92.1 (1 df)	<0.001
Clinical covariates + NLR	12	-30627.6	61279.2	117.0 (1 df)	<0.001
Clinical covariates + BMI + albumin + NLR	14	-30568.9	61165.8	234.3 (3 df)	<0.001

All models used the same 11,890 patients and 3,380 AKI events. Clinical covariates are the M2 adjustment variables defined in Table S2; each added marker was log2-transformed. Parameter counts refer to fitted covariate coefficients. Each displayed LR test compares that row with the clinical-covariates-only model, not with the ALI model. The nested ALI-versus-free-components constraint test is reported in main Table 3. Lower AIC indicates better in-sample fit; these comparisons do not establish external predictive performance or clinical incremental value. AIC, Akaike information criterion; AKI, acute kidney injury; ALI, advanced lung cancer inflammation index; LR, likelihood ratio; NLR, neutrophil-to-lymphocyte ratio.

Figure S1. IQR-scaled components and mutually exclusive surgery strata

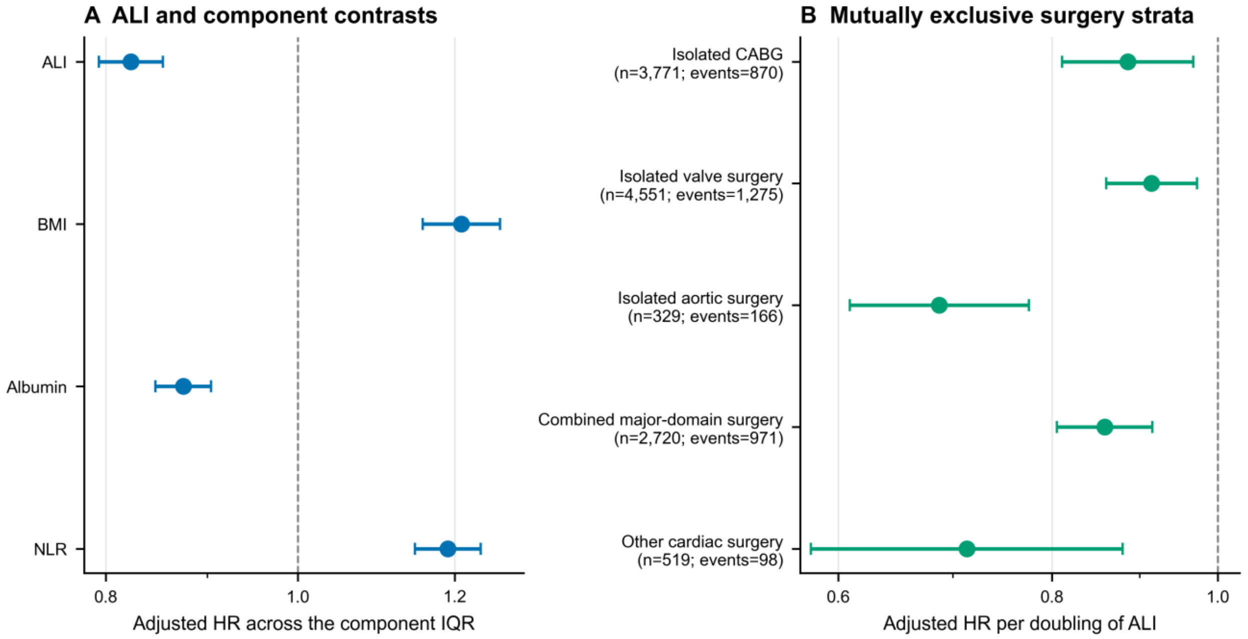

Panel A shows adjusted HRs across the observed IQR for ALI and its components. ALI was modeled with M2 covariates; BMI, albumin, and NLR were entered jointly. Panel B shows M2-adjusted HRs per doubling of ALI in mutually exclusive surgical strata. Points denote estimates and horizontal lines 95% CIs; dashed vertical lines denote HR = 1. AKI, acute kidney injury; ALI, advanced lung cancer inflammation index; BMI, body mass index; CABG, coronary artery bypass grafting; CI, confidence interval; HR, hazard ratio; IQR, interquartile range; NLR, neutrophil-to-lymphocyte ratio.

Additional supplementary analyses

The following post hoc analyses address outcome construction, differential creatinine observation, fixed-horizon AKI coding, eGFR recalculation, preoperative clinical state, procedure heterogeneity, flexible component functions, proportional-hazards diagnostics, and selection-weight balance.

Table S10. Cumulative postoperative creatinine-monitoring coverage by ALI quartile

ALI quartile	N	AKI events	Creatinine tests within 168 h, median [IQR]	Measured within 0–24 h, n (%)	Measured within 0–48 h, n (%)	Measured within 0–72 h, n (%)
Q1	2,973	1,113	5 [4–6]	2,955 (99.4%)	2,965 (99.7%)	2,969 (99.9%)
Q2	2,972	877	4 [4–5]	2,955 (99.4%)	2,968 (99.9%)	2,971 (100.0%)
Q3	2,972	722	4 [4–5]	2,955 (99.4%)	2,961 (99.6%)	2,964 (99.7%)
Q4	2,973	668	4 [3–5]	2,958 (99.5%)	2,968 (99.8%)	2,970 (99.9%)

Times are measured from surgery end. Coverage is cumulative: at least one creatinine measurement from surgery end through the indicated horizon, using the full quartile N as denominator regardless of later AKI, discharge, or death. It does not indicate a new measurement in each later interval. ALI quartiles were defined in the corrected adult cohort. The 168-hour measurement count differed across quartiles (Kruskal–Wallis $p < 0.001$). Benjamini–Hochberg adjustment covered four tests: the total-count test and the three cumulative-coverage tests. Adjusted coverage p values were 0.963, 0.308, and 0.179 at 24, 48, and 72 h, respectively. Table S14 instead uses nonoverlapping intervals and risk-set denominators. AKI, acute kidney injury; ALI, advanced lung cancer inflammation index; IQR, interquartile range.

Table S11. Fixed-horizon modified Poisson analyses for AKI within 168 hours

Analysis	N	AKI events	Adjusted RR (95% CI)	p value
Full cohort	11,890	3,380	0.86 (0.83–0.88)	<0.001
Exclude follow-up ending before 168 h without recorded AKI	11,290	3,380	0.86 (0.84–0.89)	<0.001

Modified Poisson regression used a log link and HC0 robust standard errors; RRs are per ALI doubling and adjusted for M2 covariates. The full-cohort model coded patients without recorded AKI as non-events, including those whose observation ended early; it does not establish complete post-discharge ascertainment. The follow-up-restricted sensitivity analysis excluded 600 patients whose follow-up ended before 168 h without recorded AKI, including 41 deaths (Table S13). AKI, acute kidney injury; ALI, advanced lung cancer inflammation index; CI, confidence interval; RR, risk ratio.

Table S12. Severe-AKI follow-up sensitivity analysis

Analysis	N	Severe AKI events	Excluded for incomplete follow-up	Adjusted OR (95% CI)	p value
Full cohort supportive analysis	11,890	746	0	0.67 (0.63–0.73)	<0.001
Exclude death or discharge before 168 h without severe AKI	11,172	746	718	0.67 (0.62–0.73)	<0.001

Severe AKI was the maximum recorded KDIGO stage 2 or 3 within 168 h after surgery end. Binomial logistic models used M2 covariates; ORs are per ALI doubling. The full-cohort analysis coded patients without documented severe AKI as non-events. The sensitivity analysis excluded 718 patients who died or were discharged before 168 h without severe AKI. These are odds ratios, not hazard ratios. AKI, acute kidney injury; ALI, advanced lung cancer inflammation index; CI, confidence interval; KDIGO, Kidney Disease: Improving Global Outcomes; OR, odds ratio.

Table S13. Outcome construction and fixed-horizon coding audit

Domain	Category or measure	N or value
Baseline creatinine source	Time-stamped laboratory-system baseline	11,831
Baseline creatinine source	Wide-table fallback baseline	59
Baseline creatinine timing	Timed baseline look-back, median hours	100.2
Baseline creatinine timing	Timed baseline look-back, 25th percentile hours	55.7
Baseline creatinine timing	Timed baseline look-back, 75th percentile hours	147.6
Baseline creatinine timing	Timed baseline measurements after surgery end	0
Maximum KDIGO stage within 168 h	Stage 0	8,510
Maximum KDIGO stage within 168 h	Stage 1	2,634
Maximum KDIGO stage within 168 h	Stage 2	451
Maximum KDIGO stage within 168 h	Stage 3	295
Stage at first AKI detection	Stage 1	3,105
Stage at first AKI detection	Stage 2	180
Stage at first AKI detection	Stage 3	95
First AKI event source	Serum-creatinine criterion (laboratory records)	3,369
First AKI event source	Kidney replacement therapy (stored protocol)	11
Kidney replacement therapy	Any postoperative KRT within 168 h	81
Early postoperative detection	AKI detected within 0–6 h	568
Early postoperative detection	Stored pre-6-hour-landmark AKI flag	532
Modified Poisson coding	Observed AKI	3,380
Modified Poisson coding	No recorded AKI	8,510
Modified Poisson coding	Discharge/follow-up end before 168 h without recorded AKI	600
Modified Poisson coding	Death before AKI within 168 h	41
Modified Poisson coding	Death and discharge/follow-up-end overlap	41
Modified Poisson coding	No recorded AKI with observation through 168 h	7,910

The source pipeline recorded the selected baseline, first qualifying event source and stage, maximum KDIGO stage, and postoperative KRT. Baseline look-back is surgery-end time minus stored baseline-creatinine time, in hours; the after-surgery-end count does not establish timing relative to surgery start. The pre-landmark flag refers to the postoperative landmark and is not a preoperative-AKI diagnosis. The complete creatinine-value trajectory and upstream derivation program were not retained in the locked analysis extract; the distributed files cannot independently rerun every KDIGO comparison. The 600 shorter follow-up records include the 41 deaths because death and follow-up-end timestamps coincided; these overlapping rows should not be added. AKI, acute kidney injury; KDIGO, Kidney Disease: Improving Global Outcomes; KRT, kidney replacement therapy.

Table S14. Nonoverlapping postoperative creatinine-monitoring intervals by ALI quartile

Panel A. Creatinine measurements by interval and ALI quartile

Interval	ALI quartile	At risk at start	AKI events	Tests, median [IQR]	Measured, n (%)	Observed through end	Measured through-end cohort, n (%)
0–24 h	Q1	2,973	769	2 [2–2]	2,955 (99.4%)	2,198	2,180 (99.2%)
0–24 h	Q2	2,972	623	2 [2–2]	2,955 (99.4%)	2,341	2,324 (99.3%)
0–24 h	Q3	2,972	544	2 [2–2]	2,955 (99.4%)	2,427	2,410 (99.3%)
0–24 h	Q4	2,973	506	2 [2–2]	2,958 (99.5%)	2,464	2,449 (99.4%)
>24–48 h	Q1	2,198	267	1 [1–1]	1,670 (76.0%)	1,928	1,403 (72.8%)
>24–48 h	Q2	2,341	170	1 [0–1]	1,665 (71.1%)	2,170	1,495 (68.9%)
>24–48 h	Q3	2,427	125	1 [0–1]	1,716 (70.7%)	2,301	1,591 (69.1%)
>24–48 h	Q4	2,464	106	1 [0–1]	1,733 (70.3%)	2,357	1,627 (69.0%)
>48–72 h	Q1	1,928	26	1 [0–1]	1,070 (55.5%)	1,898	1,043 (55.0%)
>48–72 h	Q2	2,170	32	1 [0–1]	1,115 (51.4%)	2,133	1,083 (50.8%)
>48–72 h	Q3	2,301	21	0 [0–1]	1,103 (47.9%)	2,280	1,082 (47.5%)
>48–72 h	Q4	2,357	19	0 [0–1]	1,154 (49.0%)	2,337	1,135 (48.6%)
>72–168 h	Q1	1,898	51	1 [1–2]	1,482 (78.1%)	1,758	1,377 (78.3%)
>72–168 h	Q2	2,133	52	1 [0–2]	1,554 (72.9%)	1,941	1,410 (72.6%)
>72–168 h	Q3	2,280	32	1 [0–2]	1,643 (72.1%)	2,102	1,515 (72.1%)
>72–168 h	Q4	2,337	37	1 [0–2]	1,632 (69.8%)	2,109	1,465 (69.5%)

Panel B. Global tests of between-quartile differences

Interval	Outcome	Test	Statistic	df	BH-adjusted p value
0–24 h	At least one creatinine measurement among patients at risk at interval start	Pearson chi-square	0.29	3	0.963
0–24 h	Creatinine measurement count among patients at risk at interval start	Kruskal-Wallis	3.23	3	0.408
>24–48 h	At least one creatinine measurement among patients at risk at interval start	Pearson chi-square	23.52	3	<0.001
>24–48 h	Creatinine measurement count among patients at risk at interval start	Kruskal-Wallis	25.82	3	<0.001
>48–72 h	At least one creatinine measurement among patients at risk at interval start	Pearson chi-square	28.05	3	<0.001
>48–72 h	Creatinine measurement count among patients at risk at interval start	Kruskal-Wallis	30.02	3	<0.001
>72–168 h	At least one creatinine measurement among patients at risk at interval start	Pearson chi-square	37.78	3	<0.001
>72–168 h	Creatinine measurement count among patients at risk at interval start	Kruskal-Wallis	86.47	3	<0.001

Panel A. Intervals are measured from surgery end. At risk at start denotes patients alive, in hospital, and without recorded AKI at the interval boundary. The measured percentage uses this at-start N; the final percentage uses the observed-through-end N. Quartiles remain those of the full cohort. Counts within an interval may include tests obtained after AKI detection in that interval; restricting to patients observed and event-free through interval end reduces but cannot fully remove this limitation. These interval-specific percentages differ from the cumulative full-cohort percentages in Table S10. AKI, acute kidney injury; ALI, advanced lung cancer inflammation index; IQR, interquartile range.

Panel B. These global between-quartile tests use the at-risk-at-start populations. Pearson chi-square tests compare measurement coverage; Kruskal–Wallis tests compare measurement counts. Benjamini–Hochberg adjustment was applied across the eight interval-specific comparisons, separately from the cumulative-monitoring tests in Table S10. BH, Benjamini–Hochberg.

Table S15. eGFR recalculation and same-frame sensitivity analyses

Panel A. eGFR recalculation audit

Recalculation audit	Value
Source eGFR missing N	82
Recalculated eGFR missing N	0
Median absolute difference among paired values	1.41
95th percentile absolute difference among paired values	4.05
Discordant classification at eGFR 60 among paired values	82
Overall eGFR median	94.70
Overall eGFR 25th percentile	79.15
Overall eGFR 75th percentile	103.09
Signed SMD for AKI minus no-AKI groups	-0.53

Panel B. Additional sensitivity models

Analysis	N	AKI events	Adjusted HR (95% CI)	p value
Primary M2	11,890	3,380	0.83 (0.80–0.86)	<0.001
M2 without documented heart failure	11,890	3,380	0.83 (0.80–0.86)	<0.001
M2 on the LVEF-complete frame	8,995	2,591	0.82 (0.79–0.86)	<0.001
M2 plus LVEF on the same complete-case frame	8,995	2,591	0.83 (0.80–0.87)	<0.001
Recalculated eGFR at least 60	10,728	2,739	0.78 (0.75–0.82)	<0.001
M2 plus available preoperative clinical-state indicators	11,890	3,380	0.90 (0.87–0.94)	<0.001

Panel A. eGFR was recalculated with the 2021 CKD-EPI creatinine equation from corrected age, sex, and baseline creatinine. The overall median [IQR] was 94.70 [79.15–103.09] mL/min/1.73 m². CKD-EPI, Chronic Kidney Disease Epidemiology Collaboration; eGFR, estimated glomerular filtration rate; SMD, standardized mean difference.

Panel B. The two LVEF rows use the same 8,995 complete cases before and after LVEF adjustment. The preoperative clinical-state model added cardiogenic shock, cardiopulmonary resuscitation, tracheal intubation, infective endocarditis, acute aortic dissection or intramural hematoma, and intravenous catecholamine use. AKI, acute kidney injury; CI, confidence interval; HR, hazard ratio; LVEF, left ventricular ejection fraction; M2, primary multivariable model.

Table S16. Procedure heterogeneity, other-surgery composition, and preoperative clinical state

Panel A. Global procedure-category interaction

Test	N	AKI events	Reference	LR χ^2	df	p value
Global ALI-by-procedure-category interaction	11,890	3,380	Isolated valve surgery	43.93	7	<0.001

Panel B. Composition of other cardiac surgery

Category	N	AKI events	AKI (%)
Other operation without CABG, valve, aortic, congenital, or transplant indicator	495	84	17.0%
Heart transplant	19	13	68.4%
Congenital surgery	5	1	20.0%

Panel C. Preoperative clinical-state indicators

Indicator	Exposed N	AKI events	Minimum exposure-by-AKI cell, n
preoperative cardiogenic shock	92	54	38
preoperative cardiopulmonary resuscitation	44	29	15
preoperative tracheal intubation	65	39	26
infective endocarditis	172	58	58
acute aortic dissection or intramural hematoma	355	234	121
preoperative intravenous catecholamine	82	46	36

Panel A. The global interaction is a joint likelihood-ratio test of all seven nonreference category-specific ALI slopes, using isolated valve surgery as the reference. It is distinct from the binary combined-versus-noncombined interaction in Table S3. ALI, advanced lung cancer inflammation index; AKI, acute kidney injury; LR, likelihood ratio.

Panel B. This category had no CABG, valve, or aortic major-domain indicator. The rows partition this category using the available congenital and transplant indicators; the residual row is defined by absent indicators and does not establish a specific operation type. Congenital and transplant indicators remained separate covariates in stratum-specific models. AKI, acute kidney injury; CABG, coronary artery bypass grafting.

Panel C. Each indicator had nonzero counts in all four exposure-by-AKI cells; the minimum cell count is reported. This marginal count check is not a formal assessment of multivariable quasi-complete separation. Adding all six indicators to M2 yielded an ALI HR of 0.90 (95% CI, 0.87–0.94; $p < 0.001$). This post hoc model assessed attenuation and was not interpreted as complete confounding control. AKI, acute kidney injury; ALI, advanced lung cancer inflammation index; CI, confidence interval; HR, hazard ratio.

Table S17. Flexible component analyses and ALI spline knots

Panel A. BMI functional-form analyses

BMI analysis	IQR contrast	Adjusted HR (95% CI)	IQR-contrast p value	Nonlinearity LR χ^2	Nonlinearity p value
Linear log2 BMI IQR contrast	22.04–26.35 kg/m²	1.21 (1.16–1.26)	<0.001	22.46 (3 df)	<0.001
Flexible BMI spline IQR contrast	22.04–26.35 kg/m²	1.21 (1.12–1.30)	<0.001	22.46 (3 df)	<0.001

Panel B. Albumin–NLR interaction

Analysis	Contrast	Estimate or test	p value
Tensor-product interaction likelihood-ratio test	Full interaction model vs additive spline model	LR χ^2 = 35.62 (9 df)	<0.001
High albumin and low NLR	Relative to median albumin and median NLR	HR 0.80 (0.76–0.85)	<0.001
Low albumin and high NLR	Relative to median albumin and median NLR	HR 1.27 (1.19–1.35)	<0.001
Low albumin and high NLR vs high albumin and low NLR	Direct joint IQR contrast	HR 1.58 (1.44–1.74)	<0.001

Panel C. ALI spline knots

Knot	Type	log2 ALI	ALI
1	Boundary	-0.093	0.94
2	Interior	4.848	28.81
3	Interior	5.402	42.30
4	Interior	5.887	59.18
5	Boundary	8.604	389.02

Panel A. Both models included albumin, NLR, and M2 covariates. The linear model used log2 BMI; the flexible model used a centered natural cubic spline of log2 BMI with four degrees of freedom. Each HR compares the displayed IQR endpoints within its own fitted model. The nonlinearity statistic is one likelihood-ratio comparison of the flexible and linear models, repeated in both rows for reference. BMI, body mass index; CI, confidence interval; HR, hazard ratio; IQR, interquartile range; LR, likelihood ratio; NLR, neutrophil-to-lymphocyte ratio.

Panel B. High and low denote the observed 75th and 25th percentiles: albumin 42.28 and 37.60 g/L and NLR 3.23 and 1.68. Joint contrasts were estimated from the tensor-product model. The first two contrasts use median albumin and median NLR as reference; the final contrast directly compares the two specified profiles. The likelihood-ratio test compares the full tensor-interaction model with the additive-spline model. These are adjusted associations, not causal interaction effects. CI, confidence interval; HR, hazard ratio; IQR, interquartile range; LR, likelihood ratio; NLR, neutrophil-to-lymphocyte ratio.

Panel C. Patsy cr(x, df = 4, constraints = 'center') generated five knots; the first and last were boundary knots. ALI, advanced lung cancer inflammation index.

Table S18. Proportional-hazards and selection-weight diagnostics

Panel A. Proportional-hazards tests

Term	χ^2	df	p value
ALI (log2)	6.71	1	0.010
Age (per 10 years)	173.83	1	<0.001
Female sex	39.27	1	<0.001
Baseline creatinine (log2)	12.03	1	<0.001
Diabetes	41.08	1	<0.001
Hypertension	4.56	1	0.033
Documented heart failure	0.06	1	0.813
CABG-containing surgery	73.36	1	<0.001
Valve-containing surgery	50.96	1	<0.001
Aortic surgery	49.87	1	<0.001
Congenital surgery	0.68	1	0.411
Heart transplantation	3.99	1	0.046
Global test	313.04	12	<0.001

Panel B. Selection-weight balance

Variable	Target mean	Selected mean	Weighted mean	SMD before	SMD after
Age, years	61.523	61.689	61.520	0.014	-0.000
Baseline creatinine (log2)	6.227	6.223	6.227	-0.009	-0.001
Female sex	0.370	0.375	0.371	0.010	0.002
Diabetes	0.269	0.273	0.268	0.010	-0.001
Hypertension	0.533	0.523	0.530	-0.021	-0.007
Documented heart failure	0.727	0.731	0.717	0.008	-0.021
CABG-containing surgery	0.430	0.440	0.431	0.020	0.002
Valve-containing surgery	0.598	0.608	0.603	0.021	0.010
Aortic surgery	0.180	0.155	0.178	-0.067	-0.005
Congenital surgery	0.005	0.005	0.005	0.000	0.000
Heart transplantation	0.005	0.002	0.003	-0.052	-0.028
Baseline creatinine missing	0.062	0.000	0.000	-0.364	-0.364

Panel A. The standard cox.zph test used the Kaplan–Meier time transform. The ALI and global tests rejected proportionality; piecewise cause-specific estimates are therefore reported in the main manuscript and Table S5. ALI, advanced lung cancer inflammation index.

Panel B. The target comprised 13,502 adult nondialysis index patients; 11,890 entered the analysis. Missing predictors were median-imputed with missingness indicators in the selection model. Values are means for continuous variables and proportions for binary indicators. SMDs compare the selected cohort with the target before and after weighting. Absolute weighted SMDs were below 0.03 except for the baseline-creatinine-missingness indicator, which remained imbalanced because every selected patient had a valid baseline value. SMD, standardized mean difference.

Figure S2. Scaled Schoenfeld residuals for log2 ALI

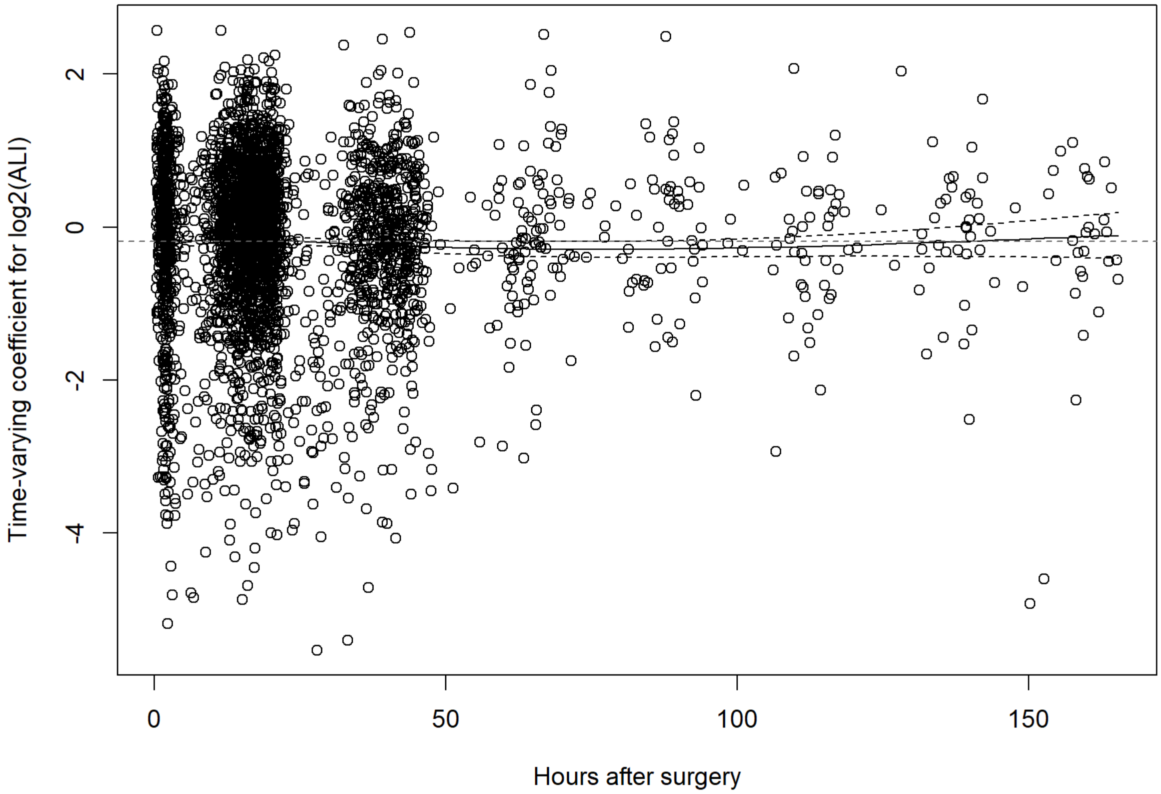

Circles show scaled Schoenfeld residuals; the solid curve shows the smoothed time-varying coefficient, dashed curves the approximate confidence band, and the horizontal dashed line the fitted average coefficient. The standard test for log2 ALI was $\chi^2 = 6.71$ ($p = 0.010$), and the global test was $\chi^2 = 313.04$ with 12 df ($p < 0.001$). ALI, advanced lung cancer inflammation index.

Supplementary Code S1

Supplementary Code S1 contains the Python and R scripts used for cohort preparation, primary analyses, and analyses added during revision, together with supporting aggregate outputs and figure-source files. The actual source-code files are supplied in the Supplementary_Code_S1 folder within the accompanying jcdd-4518666-Supplementary Material.zip archive.

Files and workflow

Open Supplementary_Code_S1_README.md in that folder for the workflow, required inputs, and example commands. The same code is also available in the separately supplied jcdd-4518666_Supplementary_Code_S1.zip archive. The principal scripts are listed below.

Cohort preparation and primary analyses: prepare_corrected_adult_cohort.py; run_association_analysis.py.

Reviewer-requested and follow-up analyses: run_reviewer_requested_analyses.py; run_integrated_revision_analyses.py; run_methodological_review_analyses.py.

Weighted Cox regression and proportional-hazards diagnostics: fit_selection_weighted_cox.R; run_additional_cox_diagnostics.R.

ALI component-constraint test: test_ali_component_constraint.py.

Observation-process summary: summarize_aki_observation_process.py.

Figure generation and style helpers: make_figure3_component_surface.py; make_r1_figure3.py; make_revised_figures.py; rebuild_final_figures_1_2_4.py; publication_style.py.

Software and required inputs

Software information is recorded in software_versions.json. The original primary-analysis environment is recorded separately in the software block of figure_source_inputs/analysis_results.json; the primary and revision analyses used different environments.

Patient-level clinical datasets are not included. Model scripts require the institutionally governed cohort extracts described in the README. The code begins with outcome-coded extracts and does not independently reconstruct every original KDIGO comparison from complete clinical records.
