# Supplementary material for: Preoperative Advanced Lung Cancer Inflammation Index and Acute Kidney Injury After Adult Cardiac Surgery: A Retrospective Cohort Study
Source: J Cardiovasc Dev Dis. 2026 Sep 30;13(10):490. doi: 10.3390/jcdd13100490 (PMC13705152; doi:10.3390/jcdd13100490)
Supplement: Supplementary file 1 [file jcdd-13-00490-s001.zip › Supplementary_Code_S1/figure_s2_scaled_schoenfeld_ali.pdf]

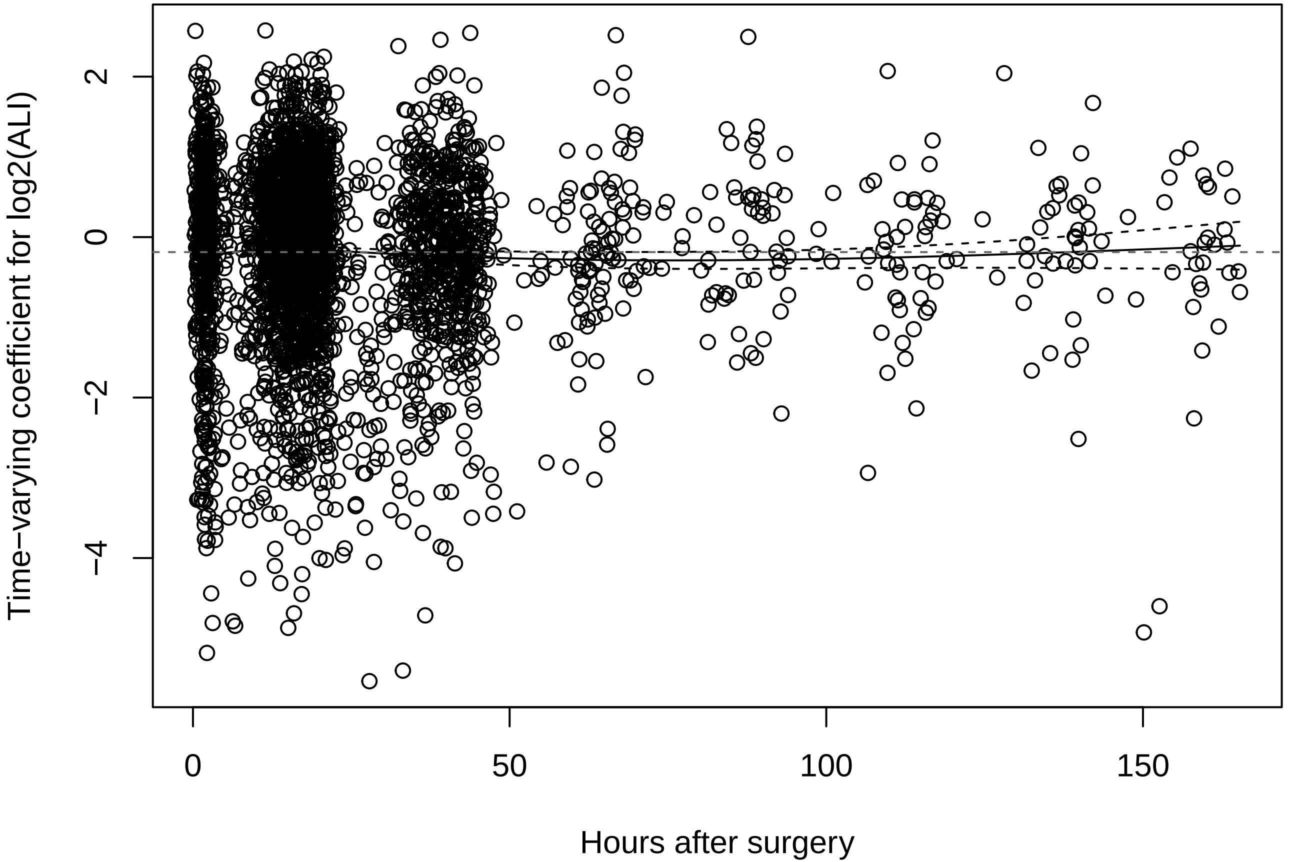
Time−varying coefficient for log2(ALI)
2
0
−2
−4
0
50
100
150
Hours after surgery
